# Risk factors and correlates for anemia in HIV treatment-naïve infected patients: a cross-sectional analytical study

**DOI:** 10.1186/1756-0500-3-230

**Published:** 2010-08-20

**Authors:** José A Mata-Marín, Jesús E Gaytán-Martínez, Rosa E Martínez-Martínez, Carla I Arroyo-Anduiza, José L Fuentes-Allen, Moisés Casarrubias-Ramirez

**Affiliations:** 1 Infectious Diseases Department, Hospital de Infectología, "La Raza" National Medical Center, IMSS, Mexico City, México; 2 Internal Medicine Department, Hospital de Especialidades, "La Raza" National Medical Center, IMSS, Mexico City, México; 3 Clinical Pathology Department, Hospital General, "La Raza" National, Medical Center, IMSS, Mexico City, México

## Abstract

**Background:**

Hematologic manifestations of the human immunodeficiency virus (HIV) infection are a well-recognized complication of the disease and may be clinically important. Our objective was to determine the risk factors for anemia and its correlation with HIV treatment-naïve infected patients without co-infection or opportunistic diseases.

**Findings:**

We performed a cross-sectional comparative study in which HIV treatment-naïve infected patients with anemia were compared with a control group of HIV patients without anemia. The interrelationship between risk factors and anemia was determined. Odds ratio and 95% confidence intervals were calculated, to adjust for the effects of potential confounders and we used a logistic regression model. Pearson's correlation coefficient was obtained to calculate the correlation between risk factors and hemoglobin.

We enrolled 54 men and 9 women. Anemia was found in 13 patients; prevalence .20 (CI 95% 0.12-0.32). Severe anemia was found in only one patient (1.5%). Only CD4+ Cells Count <200 cells/mm^3 ^was associated with increased risk of anemia in the multivariate analysis. There was a moderately strong, positive correlation between WBC and hemoglobin (r = 0.49, P < 0.001) and between CD4+ cell count and hemoglobin (r = 0.595, P < 0.001) and a moderately strong, negative correlation between HIV RNA viral load and hemoglobin (r = - 0.433, P < 0.001).

**Conclusions:**

Anemia is a common manifestation in the Mexican population without antiretroviral therapy. In HIV naïve patients a CD4+ Cell Count < 200 cells/mm^3 ^was associated with an increased risk of anemia. There is a positive correlation between hemoglobin and CD4+ cell count.

## Background

The Hematologic manifestations of the human immunodeficiency virus (HIV) infection are a well-recognized complication of the disease and may be clinically important in many patients [1,2]. An obvious cause of anemia in patients with HIV infection is blood loss. Other than blood loss, the physiopathology of HIV-associated anemia may involve three basic mechanisms: decreased RBC (Red Blood Cell) production, increased RBC destruction, and ineffective RBC production [3,4].

Although HIV associated anemia is multifactorial, the principal factors are infiltration of the bone marrow by neoplasm or infection, use of myelosuppressive medications such as zidovudine, HIV infection itself, a decreased production of endogenous erythropoietin, hemolytic anemia that may result from RBC autoantibodies, or may also develop as a consequence of the use of various medications. Anemia also may result from nutritional deficiencies--most commonly, deficiencies in iron, folic acid, or vitamin B12. In patients with HIV disease, folic acid deficiency is generally caused by either dietary deficiency or jejunal pathology. Vitamin B12 deficiency may result from malabsorption in the ileum or from gastric pathology caused by an array of infections or other conditions that affect the gastric mucosa in HIV-infected patients [5]. The association between anemia and decreased survival has been found to be independent of CD4+ T-lymphocyte count and plasma HIV RNA concentration. Anemic HIV-infected people who recover from anemia have better survival rates than those who do not recover [6-10].

Low CD4+ cells counts (<200 cells/mL) and higher HIV-1 RNA levels in plasma have each been independently associated with an increased risk of anemia in multivariate analyses, other risk factors are African American race, age, body mass index, history of pneumonia, oral candidiasis, history of fever, and zidovudine use [11,12].

The understanding of anemia causes and the strength of the relationship between the HIV viral load and risk factors have not been estimated in our population.

The purpose of the study was to determine the risk factors of anemia and the correlation in HIV naïve infected patients without co-infection or opportunistic infections.

## Material and methods

### Study population

We performed a cross-sectional comparative study in which HIV treatment-naïve infected patients with anemia were compared with a control group of HIV patients without anemia, patients were recruited prospectively. The interrelationship between each risk factor and anemia was determined.

Patients seen from March 2008 to May 2009 at the Hospital de Infectologia, "La Raza" National Medical Center, were enrolled. The ones with antiviral treatment experience, bone marrow toxic drugs use, opportunistic diseases or co-infection were excluded.

The study was approved by the hospital's ethics committee.

### Clinical data and laboratory methods

Clinical and laboratory data were collected: Blood cell count, liver function test, blood chemistry, CD4+ cell count, HIV viral load, VDRL, hepatic B surface antigen and hepatitis C status were obtained at the initial evaluation.

All patients underwent a physical examination in order to identify other causes of anemia.

Data on hemoglobin (Hb) levels, hematocrit levels, and mean corpuscular volume (MCV) were derived from samples collected at the initial baseline visit using standard techniques.

Anemia was defined as an Hb level <12 g/dl, based on standard published guidelines in women (12-14) and <14 g/dl in men. Severe anemia was defined as an Hb level <10 g/dl (15). Normal MCV was defined as 80 to 100 fl, and an MCV <80 fl or >100 fl was considered abnormal.

AIDS was defined as a self-reported history of a clinical AIDS defining condition using the 1993 Centers for Disease Control (CDC) criteria.

### Statistical methods

The odds ratio and 95% confidence intervals were calculated to assess the relationship between each risk factor and the risk of anemia; to adjust for the effects of potential confounders, we used a logistic regression model. Pearson's correlation coefficient was obtained to calculate the correlation between risk factors and hemoglobin in HIV infected adult naïve patients.

## Results

### Characteristics of the study population

Of the 82 subjects initially enrolled, 19 were excluded because of opportunistic diseases or co-infections. We enrolled 54 men and 9 women. The mean (± SD) age of our subjects was 33.65 (SD ± 9.73), hemoglobin was 14.67 g/dl (SD ± 1.799), CD4+ cell count 350 cells/ml (SD ± 285.46) and HIV viral load 223,227 copies/ml (SD ± 311,203). Clinical characteristics of the subjects evaluated in this study are presented in Table 1.

**Table 1:** Clinical characteristics in anemic and non-anemic patients

	Anemic patients (n = 13)	Non-anemic patients (n = 50)	P value
Age (years)	33.92 (± 7.27)	33.84 (± 10.33)	.765
WBC (cells/mm^3^)	4,215 (± 1,365)	6,040 (± 1,923)	**.002**
Platelets (No./mm^3^)	197,769 (± 67,551)	219,012 (± 52,330)	.225
Creatinine (mg/dl)	.98 (± .16)	.94 (± .13)	.341
Urea (mg/dl)	23.69 (± 7.89)	26.61 (± 9.35)	.307
Uric acid (mg/dl)	5.65 (± 2.01)	5.21 (± 1.64)	.412
Cholesterol (mg/dl)	133.62 (± 37.80)	148.04 (± 31.40)	.162
Triglycerides (mg/dl)	137.54 (± 44.73)	168.08 (± 97.39)	.277
AST (IU/ml)	48.77 (± 37.41)	35.22 (± 26.72)	.140
ALT (IU/ml)	52.62 (± 44.58)	41.76 (± 41.03)	.407
LDH (IU/ml)	242.62 (± 88.79)	212.30 (± 51.00)	.112
Total bilirrubins (mg/dl)	.38 (± .22)	.55 (± .36)	.107
ALP (IU/dl)	91.07 (± 27.45)	92.58 (± 44.38)	.908
Globulins (g/dl)	3.47 (± .67)	3.23 (± .88)	.363
CD4+ cells count	92 (± 108)	418 (± 279)	**.0001**
HIV RNA viral load	486,234 (± 304,850)	154,846 (± 277,001)	**.0001**

* Significant at *P *< 0.05. P-value were computed using t-test

At enrolment, hemoglobin in the anemia group patients was 11.95 (± 1.17) and in the non-anemic group was 15.37 (± 1.13). Patients with the presence of self-reported clinical AIDS was 19 (30%).

The highest factor for contracting HIV was sexual transmission in 60 (95%) of patients.

### Prevalence and risk factors for anemia

Anemia was found in 13 patients (20.3%); a prevalence of .20 (CI 95% 0.12-0.32). Severe anemia was found in only one patient (1.58%); a prevalence of .015 (CI 95% .0028-.0846).

An MCV <80 fl was found in 2 patients (15%) and was associated with anemia. Nobody had an MCV >100 fl. Eleven patients (85%) had normocytic normocromic anemia.

A univariate analysis showed that many risk factors were associated with prevalent anemia. Risk factors associated with increased risk of anemia were: WBC <4,000 cells/mm^3 ^OR 9.7 (IC95% 1.9-19.2; p = 0.07), platelets <200,000 cells/mm^3 ^OR 3.5 (IC95% 1.2-6.4; p = 0.23), CD4+ cells count <200 cells/mm^3 ^OR 8.8 (IC95% 5.3-15.8; p = 0.01) and HIV RNA viral Load >100,000 copies/ml OR 7.5 (IC95% 2.7-14.5; p = 0.01). Only CD4+ Cells Count <200 cells/mm^3 ^was associated with an increased risk of anemia in the multivariate analysis OR 16.95 (IC95% 1.7-29.6; p = 0.07).

### Correlates of anemia among HIV infected patients

Several factors were shown to be associated with anemia among HIV naïve infected patients in our study.

**Figure 1:**
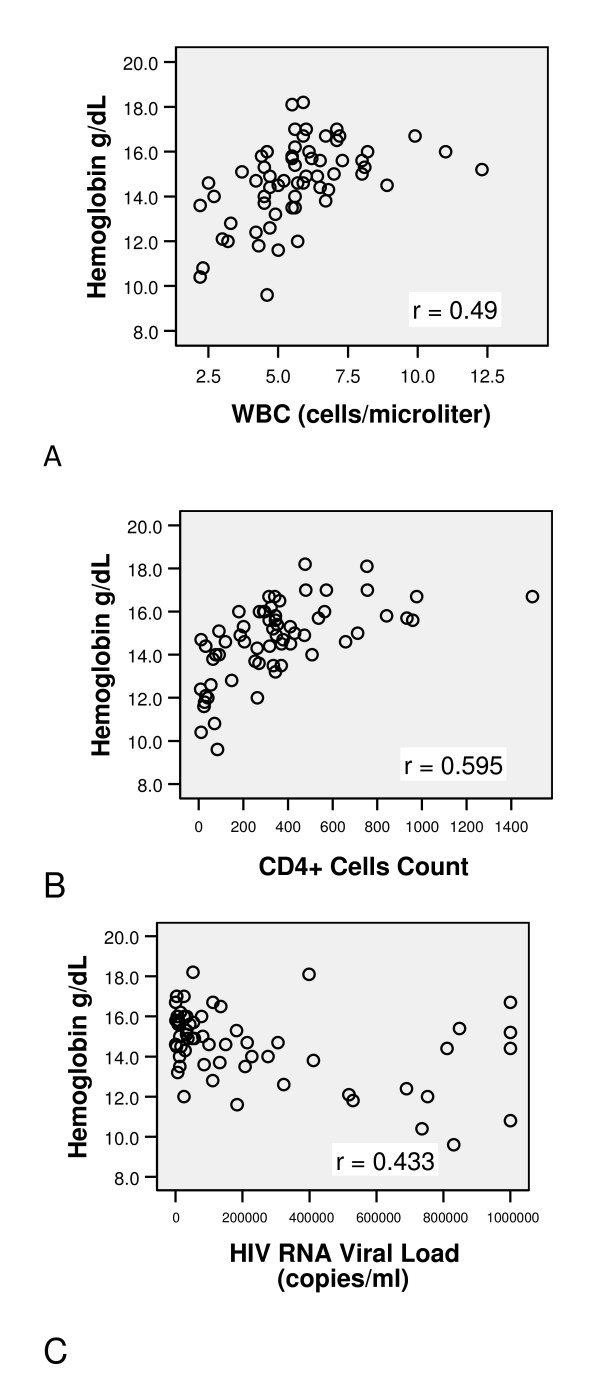
**Interrelationship between hemoglobin and risk factors**. **A**. This figure shows a moderately strong, positive correlation between WBC and hemoglobin (r = 0.49, P < 0.001). **B**. This figure shows a moderately strong, positive correlation between CD4+ cells count and hemoglobin (r = 0.595, P < 0.001). **C**. This figure shows a moderately strong, negative correlation between HIV RNA viral load and hemoglobin (r = - 0.433, P < 0.001).

We investigated the strength of the relationship between those factors that were significant in the univariate analysis, and hemoglobin levels. There was a moderately strong, positive correlation between WBC and hemoglobin (r = 0.49, P < 0.001) and between CD4+ cell count and hemoglobin (r = 0.595, P < 0.001) and a moderately strong, negative correlation between HIV RNA viral load and hemoglobin (r = - 0.433, P < 0.001). (Figure 1)

## Discussion

Although based on a small sample, we observed that anemia was related to a low CD4+ cell count (<200) as an independent factor; nevertheless, another factor was moderately strong, positively correlated with hemoglobin level, such as: WBC and CD4+ cell count; and HIV RNA viral load was moderately strong, negatively correlated. In anemic patients we had lower WBC and CD4+ cell counts compared with higher HIV RNA viral load than in the non-anemic group.

Our findings may have important biological implications, because they point out the possibility of direct bone marrow damage; in addition, these findings demonstrate that a higher HIV viral load decreased hemoglobin levels. Some authors have described abnormalities of the marrow matrix such as reticulin fibrosis, necrosis and serous atrophy of fat, These problems were observed in the bone marrow of AIDS patients. Mild to moderate myelofibrosis is probably the most common of these matrix abnormalities; fibrosis may be diffuse or focally associated with lymphoid aggregates or granulomas.^1^

We also found that normocytic normocromic anemia is the most frequent type of anemia in patients without antiretroviral experience and without co-infections or opportunistic diseases.

In their study Semba el al., concluded that anemia was in 28.1% of HIV patients, but they did not evaluate men [11]. Anemia had a higher prevalence in the Levine study (37%), but they only evaluated women; in addition, many of them (25%) were on zidovudine treatment.

Levine and colleagues reported that an inverse correlation was found between the risk of anemia and CD4 count; thus, at CD4 counts of 500 per microliter or above, 22.3% of women were anemic versus 30.4% of those with CD4 counts between 200 and 499 per microliter and 58.1% of those with CD counts <200 per microliter (p < .001). As shown, more severe levels of anemia were also found to correlate with decreasing CD4 counts [11]; in our study we correlated using hemoglobin and CD4+ cell counts, finding similar data. In the same study, HIV-1 viral load also correlated inversely with the risk of anemia (found primarily at the higher levels of HIV-1 RNA in plasma). Thus, anemia was present in 31.8% of women with HIV-1 RNA levels between 10,000 and 49,999 copies per cubic centimeter, in 43.1% of women with HIV RNA between 50,000 and 99,999 copies per cubic centimeter, and in 57.5% of those with 100,000 or more copies per cubic centimeter; ^11 ^we found a negatively strong, negative correlation between hemoglobin and HIV RNA viral load.

Risk factors for anemia such as low CD4+ cell count (<200), race (afro-american), oral candidiasis and bacterial pneumonia, were described by Semba el al., and Marieke et al., however we included patients without co-infection or opportunistic diseases, which is why we only found a low CD4+ cell count as a risk factor for anemia in our patients [12-15].

Another relatively common mechanism of anemia in HIV-infected persons has been the administration of various medications that may suppress red blood cell production [16], which is why we didn't include patients on drugs that could potentially cause bone marrow suppression.

To our knowledge, this is the first study in Mexico or Latin-America to identify the risk factors for anemia in HIV treatment-naïve infected patients; however, there were several limitations to the study. One of the most important was the small sample size; because we had to exclude many patients with antiviral treatment experience, bone marrow toxic drug use, opportunistic diseases or co-infections. In addition, this study was carried out in a single medical center: although patients were drawn from a variety of ethnic groups and socioeconomic backgrounds.

In our study we were unable to measure iron turnover, which is needed to support our clinical findings; furthermore, we didn't analyze MCV because we found <80 fl only in 2 patients and the analyses were not possible. As a result we didn't determine the etiology of the anemia.

Future studies will need to be carried out to develop interventions aimed at reducing the risk of anemia in HIV infected patients, and additional research should follow up on patients for a longer period of time.

## Conclusions

Anemia is a common manifestation in the Mexican population without antiretroviral therapy. In HIV naïve patients, a CD4+ Cell Count <200 cells/mm^3 ^was associated with an increased risk of anemia. There is a positive correlation between hemoglobin and CD4+ cell count.

## Competing interests

The authors declare that they have no competing interests.

## Authors' contributions

JAMM, JGM and REMM performed the majority of experiments. All the authors provided the collection of the human material; JAMM provided the analytic tools for this paper. JAMM, JLFA, JGM, MCR and CIAA provided vital reagents and were also involved in editing the manuscript. JAMM and JGM designed the study and all the authors wrote, read and approved the final manuscript.
